# Supplementary material for: An Agent-Based Model of Radiation-Induced Lung Fibrosis
Source: Int J Mol Sci. 2022 Nov 11;23(22):13920. doi: 10.3390/ijms232213920 (PMC9693125; doi:10.3390/ijms232213920)
Supplement: Supplementary file 1 [file ijms-23-13920-s001.zip › ijms-2002024-supplementary final/Table S1 Model parameters.pdf]

Model Parameters

Table S1. Model parameters

Name	Value	Source
activationProbability_AEC2	0.25 [day-1]	[1]
sim_time_step	1 [s]	/
sub_resolution	4	/
ECM_saturation	1e-2 [g cm-3]	[1]
$\lambda_{TGF\beta,IL13}$	1	[1]
$\lambda_{ECM,TGF\beta}$	3.65e+3	Estimated
MMP_ECM_binding	2.59e+7 [cm3 g-1 day-1]	[1]
MMP_TIMP_binding	1.04e+9 [cm3 g-1 day-1]	[1]
TIMP_MMP_binding	4.98e+8 [cm3 g-1 day-1]	[1]
ECM_flat_conc	3.26e-3 [g cm-3]	[1]
FGF2_flat_conc	0.00 [g cm-3]	[2]
IL13_flat_conc	3.20e-8 [g cm-3]	[1]
MCP1_flat_conc	0.00 [g cm-3]	[1]
MMP_flat_conc	0.37e-7 [g cm-3]	[1]
PDGF_flat_conc	0.35e-8 [g cm-3]	[1]
TGFb_ac_flat_conc	2.51e-12 [g cm-3]	[1]
TGFb_in_flat_conc	2.51e-12 [g cm-3]	Estimated
TIMP_flat_conc	5.74e-10 [g cm-3]	[1]
TNFa_flat_conc	2.50e-8 [g cm-3]	[1]
flat_conc	0.0 [g cm-3]	/
ECM_D	0.0 [µm2 day-1]	[1]
FGF2_D	5.62e+6 [µm2 day-1]	[3]
IL13_D	1.08e+6 [µm2day-1]	[1]
MCP1_D	1.73e+7 [µm2 day-1]	[1]
MMP_D	4.32e+6 [µm2 day-1]	[1]
PDGF_D	8.64e+6 [µm2 day-1]	[1]
TGFb_ac_D	4.32e+6 [µm2 day-1]	[1]
TGFb_in_D	4.32e+6 [µm2 day-1]	Estimated
TIMP_D	4.32e+6 [µm2 day-1]	[1]
TNFa_D	1.29e+6 [µm2 day-1]	[1]
ECM_d	3.70e-1 [day-1]	[1]
FGF2_d	1.66 [day-1]	[4]
IL13_d	1.25e+1 [day-1]	[1]
MCP1_d	1.73 [day-1]	[1]
MMP_d	4.32 [day-1]	[1]
PDGF_d	3.84 [day-1]	[1]
TGFb_ac_d	3.33e+2 [day-1]	[1]
TGFb_in_d	1.10e+1 [day-1]	Estimated
TIMP_d	2.16e+1 [day-1]	[1]
TNFa_d	5.55e+1 [day-1]	[1]
k_FGF2	1.72e-9 [g cm-3]	[5]
k_IL13	2.00e-7 [g cm-3]	[1]
k_MCP1	5.00e-9 [g cm-3]	[1]
k_PDGF	1.50e-8 [g cm-3]	[1]
k_TGFb_ac	1.00e-10 [g cm-3]	[1]
k_TGFb_in	1.00e-10 [g cm-3]	Estimated

$\lambda_{FGF2,TGF\beta}$	3.00e-10 [g cm-3]	[6]
k_TNFa	5.00e-7 [g cm-3]	[1]
AEC2_proliferation	2.63e-2 [day-1]	Estimated
F_proliferation_IL13_TGFb	2.50e-1 [day-1]	Estimated
$\lambda_{F,AEC2}$	1.99 [day-1]	Estimated
M0_basic_prob_production	4.38e-2 [day-1]	Estimated
AEC2_AEC1_differentiation	9.80e-3 [day-1]	Estimated
F_MF_PDGF_differentiation	1.20e-1 [day-1]	Estimated
F_MF_TGFb_ac_differentiation	1.20e-1 [day-1]	Estimated
M1_M2_differentiation	1.52e-2 [day-1]	Estimated
M2_M1_TNFa_differentiation	5.00e-3 [day-1]	[1]
AEC1_apoptosis	1.65e-2 [day-1]	Estimated
AEC2_apoptosis	1.65e-2 [day-1]	[1]
Senescent_AEC2_apoptosis	0.0 [day-1]	Estimated
F_apoptosis	1.66e-2 [day-1]	[1]
M1_apoptosis	2.00e-2 [day-1]	[1]
M2_apoptosis	1.50e-2 [day-1]	[1]
MF_apoptosis	1.66e-2 [day-1]	[1]
AEC2_FGF2_secretion	1.93e-14 [g day-1]	Estimated
AEC2_MCP1_secretion	5.60e-14 [g day-1]	Estimated
AEC2_TNFa_secretion	2.29e-11 [g day-1]	Estimated
F_TGFb_secretion	6.30e-17 [g day-1]	Estimated
F_ECM_secretion	3.50e-11 [g day-1]	Estimated
MF_ECM_secretion	7.00e-11 [g day-1]	Estimated
M1_TNFa_secretion	1.30e-15 [g day-1]	Estimated
M2_IL13_secretion	2.87e-14 [g day-1]	Estimated
M2_MMP_secretion	1.44e-12 [g day-1]	Estimated
M2_PDGF_secretion	1.24-13 [g day-1]	Estimated
M2_TGFb_secretion	9.99e-15 [g day-1]	Estimated
M2_TIMP_secretion	2.87e-13 [g day-1]	Estimated
K_damage_prob	4.18 [day-1]	[7]
Infection_radius	110.00 [μm]	Estimated
NeighboursThreshold	1	Estimated
Phagocytic_fraction	100%	Estimated
Phagocytic_index	1	[8] & Estimated
Apoptosis_prob_after_damage	0.00 [day-1]	Estimated
damage_distance	23.18 [μm]	Estimated
phagocytic_distance	15.17 [μm]	Estimated
mesenchymal_speed	240.00 [μm day-1]	[9]
macrophage_speed	5760.00 [μm day-1]	[10]
AEC1_speed	60.00 [μm day-1]	Estimated
AEC2_speed	600.00 [μm day-1]	[11]
fibr_to_center	121.49 [μm]	Estimated
macrophage_to_center	96.21 [μm]	Estimated
alveolus_radius	110.00 [μm]	[12]
int_cells_volume	774.00 [μm^3]	[13]
alv_macr_diam	16.00 [μm]	[14]
ep_2_volume	815.00 [μm^3]	[13]
ep_1_volume	2391.00 [μm^3]	[13]

1. Hao W, Marsh C, Friedman A. A mathematical model of idiopathic pulmonary fibrosis. *PLoS One* (2015) **10**:1–19. doi:10.1371/journal.pone.0135097
2. Li CM, Khosla J, Pagan I, Hoyle P, Sannes PL. TGF-β1 and fibroblast growth factor-1 modify fibroblast growth factor-2 production in type II cells. *Am J Physiol - Lung Cell Mol Physiol* (2000) **279**:1038–1046. doi:10.1152/ajplung.2000.279.6.l1038
3. Kołodziej M, Sauer DG, Beck J, Marek WK, Hahn R, Jungbauer A, Dürauer A, Piątkowski W, Antos D. Scale up of a chromatographic capture step for a clarified bacterial homogenate – Influence of mass transport limitation and competitive adsorption of impurities. *J Chromatogr A* (2020) **1618**: doi:10.1016/j.chroma.2020.460856
4. Dvorak P, Bednar D, Vanacek P, Balek L, Eiselleova L, Stepankova V, Sebestova E, Kunova Bosakova M, Konecna Z, Mazurenko S, et al. Computer-assisted engineering of hyperstable fibroblast growth factor 2. *Biotechnol Bioeng* (2018) **115**:850–862. doi:10.1002/bit.26531
5. Grazul-Bilska AT, Luthra G, Reynolds LP, Bilski JJ, Johnson ML, Adbullah SA, Redmer DA, Abdullah KM. Effects of basic fibroblast growth factor (FGF-2) on proliferation of human skin fibroblasts in type II diabetes mellitus. *Exp Clin Endocrinol Diabetes* (2002) **110**:176–181. doi:10.1055/s-2002-32149
6. Xiao L. TGF-beta 1 induced fibroblast proliferation is mediated by the FGF-2/ERK pathway. *Front Biosci* (2012) **17**:2667. doi:10.2741/4077
7. McMahon SJ, Butterworth KT, Trainor C, McGarry CK, O'Sullivan JM, Schettino G, Hounsell AR, Prise KM. A Kinetic-Based Model of Radiation-Induced Intercellular Signalling. *PLoS One* (2013) **8**:15–18. doi:10.1371/journal.pone.0054526
8. Hu B, Sonstein J, Christensen PJ, Punturieri A, Curtis JL. Deficient In Vitro and In Vivo Phagocytosis of Apoptotic T Cells by Resident Murine Alveolar Macrophages. *J Immunol* (2000) **165**:2124–2133. doi:10.4049/jimmunol.165.4.2124
9. Rikard SM, Athey TL, Nelson AR, Christiansen SLM, Lee JJ, Holmes JW, Peirce SM, Saucerman JJ. Multiscale Coupling of an Agent-Based Model of Tissue Fibrosis and a Logic-Based Model of Intracellular Signaling. *Front Physiol* (2019) **10**: doi:10.3389/fphys.2019.01481
10. Pollmächer J, Figge MT. Agent-based model of human alveoli predicts chemotactic signaling by epithelial cells during early Aspergillus fumigatus infection. *PLoS One* (2014) **9**:e111630. doi:10.1371/journal.pone.0111630
11. Legrand C, Gilles C, Zahm JM, Polette M, Buisson AC, Kaplan H, Birembaut P, Tournier JM. Airway epithelial cell migration dynamics: MMP-9 role in cell- extracellular matrix remodeling. *J Cell Biol* (1999) **146**:517–529. doi:10.1083/jcb.146.2.517
12. Ochs M, Nyengaard JR, Jung A, Knudsen L, Voigt M, Wahlers T, Richter J, Gundersen HJG. The Number of Alveoli in the Human Lung. *Am J Respir Crit Care Med* (2004) **169**:120–124. doi:10.1164/rccm.200308-1107oc
13. Stone KC, Mercer RR, Gehr P, Stockstill B, Crapo JD. Allometric relationships of cell numbers and size in the mammalian lung. *Am J Respir Cell Mol Biol* (1992) **6**:235–243. doi:10.1165/ajrcmb/6.2.235
14. Fathi M, Johansson A, Lundborg M, Orre L, Sköld CM, Camner P. Functional and morphological differences between human alveolar and interstitial macrophages. *Exp Mol Pathol* (2001) **70**:77–82. doi:10.1006/exmp.2000.2344
