# Supplementary figures and images for: An Agent-Based Model of Radiation-Induced Lung Fibrosis
Source: Int J Mol Sci. 2022 Nov 11;23(22):13920. doi: 10.3390/ijms232213920 (PMC9693125; doi:10.3390/ijms232213920)

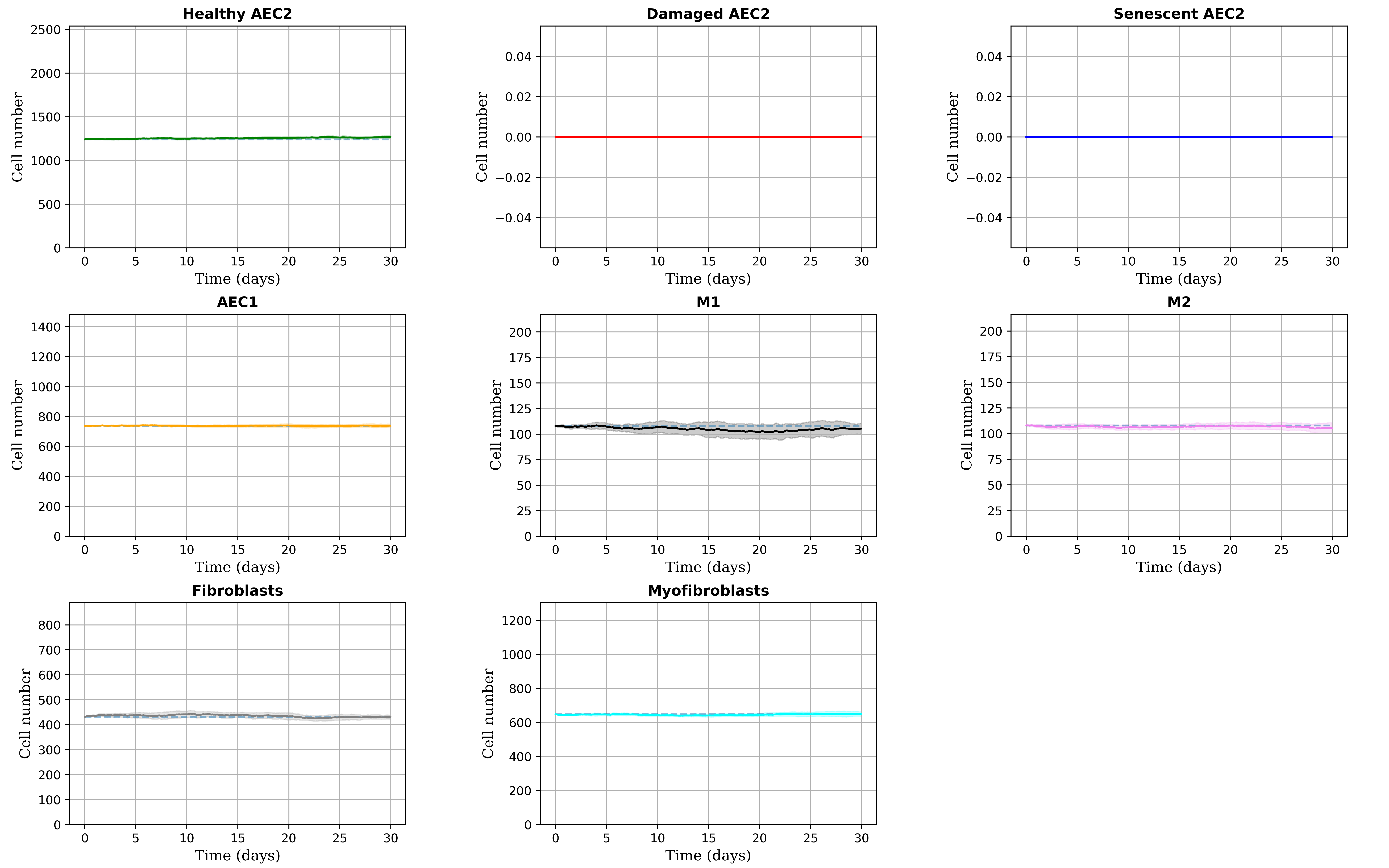

Supplement: Supplementary file 1 [file ijms-23-13920-s001.zip › ijms-2002024-supplementary final/Figure S1.png]

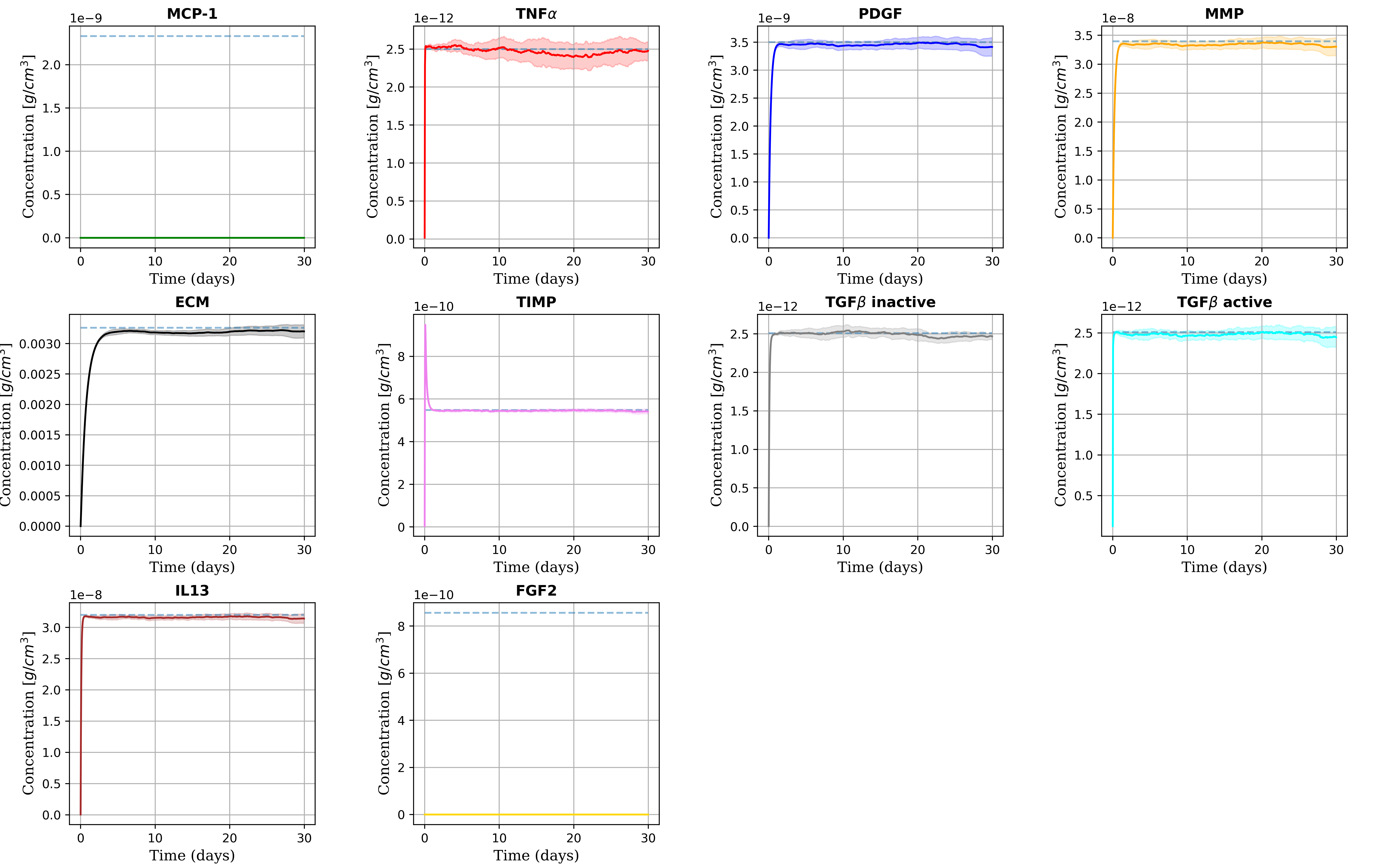

Supplement: Supplementary file 1 [file ijms-23-13920-s001.zip › ijms-2002024-supplementary final/Figure S2.png]
